# Pan-Cancer analysis shows that ACO2 is a potential prognostic and immunotherapeutic biomarker for multiple cancer types including hepatocellular carcinoma

**DOI:** 10.3389/fonc.2022.1055376

**Published:** 2022-11-30

**Authors:** Zhen Wang, Wanqun Zheng, Zhen Chen, Shilun Wu, Haoxiao Chang, Ming Cai, Heping Cai

**Affiliations:** ^1^ Department of Pharmacy, Anhui Provincial Children’s Hospital, Hefei, China; ^2^ Department of Chinese Medicine, The First Affiliated Hospital of Anhui Medical University, Hefei, Anhui Province, China; ^3^ Department of Pharmacy, The Third People’s Hospital of Hefei, Hefei, China; ^4^ Department of Hepatobiliary Surgery, Beijing Chaoyang Hospital Affiliated to Capital Medical University, Beijing, China; ^5^ China National Clinical Research Center for Neurological Disease, Beijing Tiantan Hospital, Capital Medical University, Beijing, China; ^6^ Department of Pharmacy, The Second Affiliated Hospital of Anhui University of Chinese Medicine, Hefei, China; ^7^ Anhui Acupuncture and Moxibustion Clinical Medicine Research Center, The Second Affiliated Hospital of Anhui University of Chinese Medicine, Hefei, China

**Keywords:** ACO2, pan-cancer, prognosis, immune infiltration, hepatocellular carcinoma, lipidomics

## Abstract

**Background:**

Recent evidence increasingly suggests key roles for the tricarboxylic acid cycle and fatty acid metabolism in tumor progression and metastasis. Aconitase 2 (ACO2) is a component of the tricarboxylic acid cycle and represents a key cellular metabolic hub that promotes *de novo* fatty acid biosynthesis. However, there have been few reports on the role of ACO2 in tumorigenesis and cancer progression.

**Methods:**

Through the comprehensive use of datasets from The Cancer Genome Atlas, Genotype-Tissue Expression Project, cBioPortal, Human Protein Atlas, UALCAN, Gene Expression Profiling Interactive Analysis, DNA Methylation Interactive Visualization Database, and TIMER2, we adopted bioinformatics methods to uncover the potential carcinogenic roles of ACO2, including by analysing ACO2 expression and correlations between prognosis, genetic mutations, immune cell infiltration, DNA methylation, tumor mutational burden, and microsatellite instability in different tumors. Additionally, the expression level and tumor-promoting effect of ACO2 were verified in hepatocellular carcinoma (HCC) cells. To explore the underlying mechanisms of ACO2 in human cancer, ACO2-related gene enrichment analysis and lipid metabolomics were performed using LM3 cells with or without ACO2 knockdown.

**Results:**

The results indicated that ACO2 was highly expressed in most cancers, showing early diagnostic value in six tumor types, and was positively or negatively associated with prognosis in different tumors. Moreover, ACO2 expression was associated with immune cell infiltration, such as CD8+ T cells and tumor-associated neutrophils, in some cancers. For most cancer types, there was a significant association between immune checkpoint-associated genes and ACO2 expression. Compared with normal hepatocytes, ACO2 was upregulated in HCC cells, which promoted their proliferation and migration. Furthermore, to explore the underlying molecular mechanism, we performed KEGG pathway enrichment analysis of ACO2-associated genes and lipidomics using LM3 cells with or without ACO2 knockdown, which screened 19 significantly altered metabolites, including 17 with reduced levels and 2 with increased levels.

**Conclusion:**

Through pan-cancer analysis, we discovered for the first time and verified that ACO2 could be a useful diagnostic biomarker for cancer detection. Additionally, ACO2 could be used as an auxiliary prognostic marker or as a marker for immunotherapy in some tumor types.

## Introduction

Cancer is the second leading cause of death worldwide, and it will probably become the leading cause by 2060 (1). Accumulating evidence reveals that metabolic reprogramming plays a pivotal role in tumor cell survival during metastatic dissemination, especially while cells are in circulation and colonizing distant organs (2). It is well known that cancer cells produce lactic acid from glucose even under non-hypoxic conditions, an observation that has come to be known as the Warburg effect (3), which is partly due to the defect in mitochondria TCA cycle and/or mitochondria functions resulting from the mutations of TCA cycle (4).

However, such concept has been challenged and may need to be revisited because studies have demonstrated that mitochondrial activity is fully functional in cancers on the basis of 13C-labelled metabolomic analysis, which could induce mitochondrial biogenesis and oxidative phosphorylation and promote metastasis (5). Moreover, with the discoveries and in-depth studies of oncogenic mutations in mitochondrial metabolic enzymes, such as fumarate hydratase (FH) (6), succinate dehydrogenase (SDH) (7) and isocitrate dehydrogenase 2 (IDH2) (8), it is now untenable to deny the role of TCA cycle in tumorigenesis.

As a key enzyme in the tricarboxylic acid cycle, aconitase 2 (ACO2) is closely related to mitochondrial citrate synthesis, which is the precursor metabolite required for lipogenesis and is exported to the cytosol for conversion into acetyl-CoA and subsequently into malonyl-CoA for *de novo* fatty acid biosynthesis (9). Significantly, increasing evidence shows that lipid metabolism is commonly enhanced at different stages of cancer development. These alterations go beyond energetically fueling tumor cells and trigger signaling and epigenetic events, as well as changes in membrane composition that favor metastasis (10). However, few studies on ACO2 and tumor development have been reported, mainly including prostate cancer (11), breast cancer (12), and colorectal cancer (13).

To fully assess the role of ACO2 across cancers, in this study, we tentatively performed a pan-cancer analysis that primarily involved gene expression, survival/prognosis, genetic alteration, methylation, and ACO2-associated immune infiltration data from large clinical datasets to explore the oncogenic role of ACO2. Furthermore, we confirmed the expression and tumor-promoting effect of ACO2 in hepatocellular carcinoma (HCC) cell lines, after which ACO2-related gene enrichment analysis and lipid metabolomics were performed to observe the potential underlying molecular mechanisms. Our data suggest that ACO2 could be employed as a diagnostic, prognostic, and/or immunological predictor in some cancers, which may ultimately lead to ACO2-targeting anti-tumor therapies.

## Materials and methods

### Differential expression analysis of ACO2

The mRNA expression profiles and correlative clinical data from 33 types of cancer and healthy control samples from The Cancer Genome Atlas (TCGA) and Genotype-Tissue Expression (GTEx) were extracted for analysis through their portal websites (14, 15). The whole dataset was filtered, missing and duplicated results were deleted, and the data were transformed by log2(TPM +1). The t tests were performed on the expression data and these tumor types. The expression difference between tumor and normal tissue samples was identified by the standard of P < 0.05. R software (Version 4.0.3, https://www.Rproject.org) was used for data analysis, and the “ggplot2” R package was applied to draw the box diagrams.

To further verify ACO2 protein expression, the “pathology” module of the Human Protein Atlas (HPA) online website (https://www.proteinatlas.org/) (16) was used to obtain immunohistochemical staining of ACO2 in available tumor tissues and corresponding normal tissues.

### Analysing the diagnostic value of ACO2

As provided with each sample by TCGA, specific tumor stages were chosen, and associations with ACO2 expression were analysed using “ggplot2” R packages, which are drawing packages that can separate drawing and data, data-related drawing, and data irrelevant drawing.

To evaluate the diagnostic accuracy of ACO2, using TCGA combined with GTEx cohort, receiver operating characteristic (ROC) curve analysis was conducted on the basis of sensitivity and specificity using the “pROC” package, with area under the curve (AUC) ranging from 1.0 (perfect diagnostic) to 0.5 (no diagnostic value) (17).

### Analysis of prognostic association with ACO2

The Gene Expression Profiling Interactive Analysis 2 (GEPIA 2) database (http://gepia.cancer-pku.cn) (18) is an online platform that uses a common processing technique to examine RNA sequencing expression data from the TCGA and GTEx projects. We used the Kaplan-Meier “Survival Map” module of GEPIA 2 to obtain the overall survival (OS) and disease-free survival (DFS) significance map data for ACO2 across all TCGA tumors. The log-rank test was used to test the hypothesis, and survival plots were obtained through the “Survival Analysis” module of GEPIA2.

### Genetic alteration and methylation analysis

The cBioPortal for cancer genomics (https://www.cbioportal.org/) (19) provides a web resource for exploring, visualizing, and analysing multidimensional cancer genomics data. We chose the “TCGA Pan Cancer Atlas Studies” in the “Quick select” section and entered “ACO2” to query the genetic alteration characteristics of ACO2. We collected the genetic alteration features, alteration frequencies, mutation types, copy number alteration (CNA), mutated site information, and three-dimensional (3D) structure from cBioPortal. OS, DFS, progression-free survival (PFS), and progression-free interval (PFI) data for tumors with or without ACO2 genetic alterations were collected. Log-rank p values were obtained, and Kaplan-Meier analysis was performed.

The DNA Methylation Interactive Visualization Database (DNMIVD, http://www.unimd.org/dnmivd/) (20) is a comprehensive annotation and interactive visualization database for DNA methylation profiles of diverse human cancers that was constructed from high-throughput microarray data. The mean methylation levels of ACO2 from DNMIVD in tumors were compared with corresponding normal tissues. The correlation of TREM2 methylation with prognosis was conducted using Kaplan-Meier survival analysis, including OS, DSS, DFI, and PFI (P < 0.05 as significant).

### Correlation analyses of ACO2 with tumor mutational burden and microsatellite instability

TMB is a quantifiable immune-response biomarker that reflects the number of mutations in tumor cells (21). MSI results from MMR deficiency and is associated with patient outcomes (22). Data from the Assistant for Clinical Bioinformatics (ACLBI) database (https://www.aclbi.com/static/index.html#/) (23), an online portal website for data analysis, were assessed to explore the correlation between ACO2 expression and TMB and MSI. All the analysis methods and R packages were implemented by R version 4.0.3. The two-group data were analysed by the Wilcoxon test. P < 0.05 was considered statistically significant.

### Analysis of immune infiltration

Tumor Immune Estimation Resource 2 (TIMER2.0) (http://timer.cistrome.org/) (24) provides four modules to investigate associations between immune infiltration and genetic or clinical features and four modules to explore cancer-related associations in TCGA cohorts. The TIMER, CIBERSORT, CIBERSORT-ABS, QUANTISEQ, XCELL, MCPCOUNTER, and EPIC algorithms were applied to analyse correlations between ACO2 expression and immune cell infiltration, including CD8+ T cells, CD4+ T cells, tumor-associated macrophages (TAMs), natural killer (NK) cells, tumor-associated neutrophils (TANs), and B cells. TIMER has previously computed and saved the immune cell infiltration scores of pan-cancer data from the TCGA database. The infiltration data were retrieved and examined to determine whether there was a link between ACO2 expression and infiltration. Purity-adjusted Spearman’s rank correlation tests were used to obtain p values and partial correlation values.

The “immune checkpoint” and “immunoregulation” module of SangerBox 3.0 (http://SangerBox.com/Tool) website (25, 26), a comprehensive clinical bioinformatics analysis platform, was used to explore the correlation between ACO2 expression and immunostimulators.

### ACO2-related gene enrichment analysis

The STRING website (https://string-db.org/) (27) was used to obtain ACO2-binding proteins, as demonstrated by experiments. We subsequently used the ‘Similar Gene Detection’ module of GEPIA2 to obtain the top 100 ACO2-correlated target genes in HCC and analysed correlations between ACO2 and selected genes. To perform intersection analysis of ACO2-binding and ACO2-interacting genes, Jvenn (28), an interactive Venn diagram viewer, was applied. The enrichment analyses of ACO2-related genes and proteins were performed using Kyoto Encyclopedia of Genes and Genomes (KEGG) and Gene Ontology (GO) tools in HiPlot (https://hiplot.com.cn) (29), a comprehensive web platform for scientific data visualization. The results with p<0.05 after applying the Benjamini–Hochberg correction were considered significant.

### Analysis of the single-cell RNA sequencing dataset of ACO2

The raw count and metadata of GSE1112271 (30), which contains human HCC samples, were downloaded from GEO. The R package ‘Seurat’ (31) was used to create objects and calculate the proportion of mitochondrial genes. Cells were selected based on the following criteria: cells expressing < 200 genes, nCounts_RNA < 40000 and mitochondrial genes < 10% were filtered out for exclusion of noncell or cell aggregates. Subsequently, canonical correlation analysis was performed to identify common sources of variation between the normoxia and hypoxia groups using the “RunCCA” function (32). The ‘FindAllMarkers’ function was used to identify marker genes, which were subsequently used to tag cell clusters as certain cell types (min.pct =0.25, logfc. threshold =0.25). Then, we divided the malignant cells into ACO2high cells and ACO2low cells based on the expression of ACO2 and used the ‘FindMarkers’ function to calculate the difference in gene expression between the two cell groups. For enrichment of gene set function analysis, we used Sangerbox (http://www.sangerbox.com/tool) to obtain the latest KEGG pathway gene annotation. The minimum gene was set as 40, and the maximum gene was set as 5000. A P value of < 0.05 was considered statistically significant.

### Cell culture

LO2, HepG2, LM3, and Hep1-6 cells were obtained from the cell bank of The Shanghai Institute of Biological Sciences (Shanghai, China) and maintained in DMEM supplemented with 10% fetal bovine serum at 37°C and 5% CO2. All cell lines were routinely monitored for mycoplasma (4A Biotech Co, Beijing, China).

### RNA isolation and quantitative real-time PCR

Cells were lysed using TRIzol reagent (Invitrogen, Waltham, MA, USA), and total RNA was extracted with chloroform and isopropyl alcohol. cDNA was then synthesized using a Reverse Transcription Kit (TaKaRa, Kusatsu, Japan) in accordance with the manufacturer’s protocols. A SYBR Green Master Mix Kit was used for the relative quantification of RNA levels in accordance with the manufacturer’s instructions. GAPDH was chosen as the internal control. ACO2 expression levels were normalized to the internal controls and determined by the 2−ΔΔCt method. The sequences of the primers used for qPCR were as follows: β-actin (control): forwards, 5′-CCTAGAAGCATTTGCGGTGG-3′ and reverse, 5′-GAGCTACGAGCTGCCTGACG-3′; and ACO2: forwards, 5′-CAATCGTCACCTCCTACAACAGG-3′ and reverse, 5′-GTCTCTGGGTTGAACTTGAGGG-3′.

### Western blot analysis

RIPA Lysis and Extraction Buffer (Thermo Fisher Scientific, Waltham, MA, USA) was used for total protein extraction. Equal amounts of total protein samples were separated by SDS−PAGE and then transferred to polyvinylidene fluoride membranes (Millipore, Burlington, MA, USA). Anti-ACO2 primary antibody (ab129069, Abcam, Cambridge, UK) was incubated with the membranes at 4°C overnight. After washing, the membranes were incubated with the secondary antibody at room temperature for 1 h. Immunoreactive bands were visualized using an ECL kit. Relative expression from western blot results was calculated by the amount target protein/β-actin using Intensity of Opacity (IOD) data from ImagePro Plus.

### Wound-healing assay

Cells were seeded in 6-well plates at a high density and allowed to form cell monolayers overnight. A 200-μL sterile plastic tip was used to create a wound line across the surface of the wells, and cells in suspension were removed by washing with PBS. Cells were cultured in reduced serum DMEM in a humidified 5% CO2 incubator at 37°C for 48 h, and then images were taken with a phase-contrast microscope. Each assay was replicated three times.

### Cell counting Kit-8 assay

We used the CCK-8 assay (Dojindo Molecular Technologies, Rockville, MD, USA) to measure the proliferation of HCC cells. The assay was performed by initially plating 2×10^3^ cells per well, in accordance with the manufacturer’s protocol. We then performed the assay at 0 h, 24 h, 48 h, 72 h, and 96 h.

### Transwell migration assay

For Transwell migration assays, 2.5×10^4^ transfected cells were seeded into the upper chamber with serum-free medium, while the bottom chamber contained DMEM with 10% FBS. After the cells had migrated for 24 h, they were fixed and stained with crystal violet. Migrated HCC cells were counted under an inverted light microscope.

### Establishment of stable knockdown cell lines

For knockdown of ACO2, shRNA sequences targeting ACO2 (5’-GCCCAACGAGTACATCCATTA-3’) were inserted into the LV3-pGLV-H1-GFP/puro lentiviral vector (GenePharma, Suzhou, China). The empty vector was used as a negative control. The cells were infected with lentivirus supernatant and selected in medium containing puromycin (2 μg/ml).

### Lipidomic analysis by liquid chromatography−mass spectrometry

After washing twice with PBS, cells (approximately 107 cells/ml) were suspended in 1 ml normal saline. The cell suspension was centrifuged at 4°C and 1000 rpm/min for 3 min. After centrifugation, the cells were resuspended in 0.1 mL water in a 1.5 mL centrifuge tube, followed by the addition of 0.4 ml isopropyl alcohol. The internal standard (d4-LCA and d8-phenylalanine) was added at a concentration of 5 μM. After 3 min of vortexing, the mixture was centrifuged at 15000 rpm/min for 15 min. Then, the supernatants were used for LC−MS analysis.

For lipidomics measurement, supernatants were analysed by injection onto an ACQUITY UPLC BEH C8 column (130 Å, 1.7 µm, 2.1 mm X 100 mm, 1/pk; Waters, America) at a flow rate of 0.3 ml/min using an LC-30AD Shimadzu pump system and an SIL-20AXR autosampler interfaced with an API 6500Q-TRAP mass spectrometer (SCIEX, Framingham, MA). A discontinuous gradient was generated to resolve the analytes by mixing solvent A (methol/acetonitrile/water=1/1/1 with 5 mM ammonium acetate) with solvent B (isopropyl alcohol with 5 mM ammonium acetate). A gradient elution was optimized for the separation: 0-1 min, 20% Solution B; 1-2.5 min, 20%-40% Solution B; 2.5-4.0 min, 40%-60% Solution B; 4.0-14.0 min, 60%-90% Solution B; 14.0-15.0 min, 90% Solution B; 15.0-15.1 min, 90-20% Solution B; 15.1-17 min, 20% Solution B, with a total run time of 17 mins. The flow rate was 0.3 ml/min, and the column was maintained at 40°C. Analytes were monitored using electrospray ionization in separately positive-ion mode and negative-ion mode with multiple reaction monitoring (MRM) of precursor and characteristic product-ion transitions.Raw data were acquired using Xcalibur 4.1 (Thermo Fisher Scientific), and lipids were identified with 5 ppm mass tolerance by LipidSearch 4 (Thermo Fisher Scientific). Orthogonal partial least squares-discriminant analysis (OPLS-DA) was used to identify the differential metabolites between two groups using SIMCA-P version 14 (Umetrics, Umeå, Sweden).

### Statistical analysis

For bioinformatic validation, all the gene expression data were normalized by log2 transformation. The link between ACO2 expression and targets of interest, such as immune cell infiltration scores, TMB and MSI, was assessed using Spearman’s or Pearson’s test. The Kaplan-Meier curve, log-rank test and Cox proportional hazard regression model were used for all survival analyses in this study. Depending on whether the samples were paired, paired t tests or the t test were used to compare ACO2 expression levels across groups or between tumor and normal tissues. Significance was defined as a P value of less than 0.05. All statistical analyses were processed by R software (Version 4.0.3).

For molecular biology verification, all statistical calculations were performed using the GraphPad Prism (GraphPad Software Inc., San Diego, California, USA). Data are reported as the means ± SD. Two-tailed Student’s t test was used to determine statistically significant differences. Differences were defined as statistically significant if the P value <0.05.

## Results

### Gene expression analysis and the diagnostic value of ACO2

**Figure 1:**
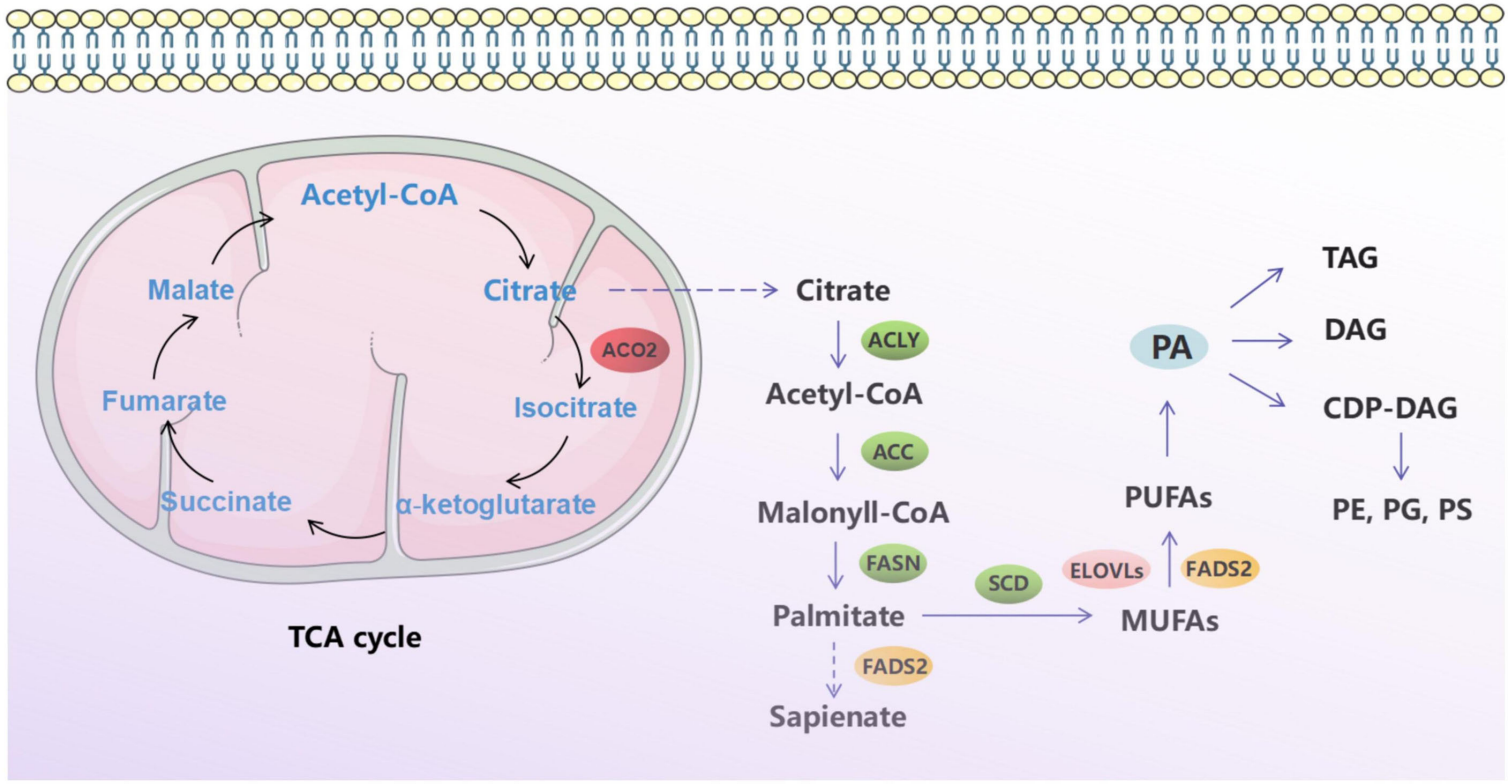
Schematic diagram of tricarboxylic acid cycle and fatty acid metabolism.

**Figure 2:**
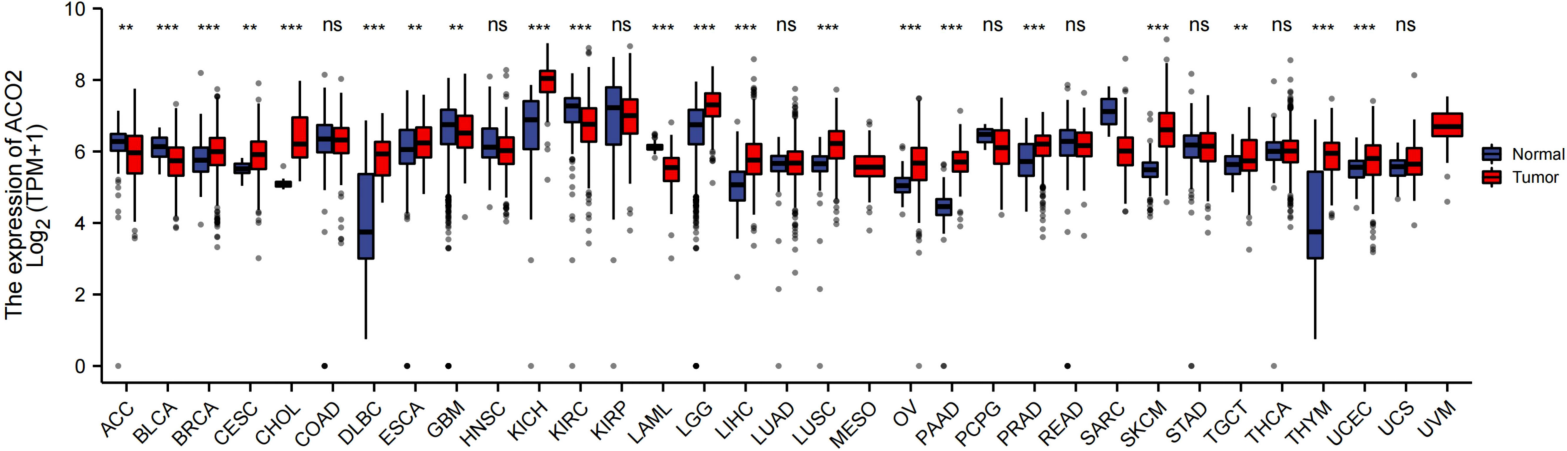
Comparison of ACO2 expression between tumor and normal samples. **p < 0.01, ***p < 0.001, ns, not statistically significant.

**Figure 3:**
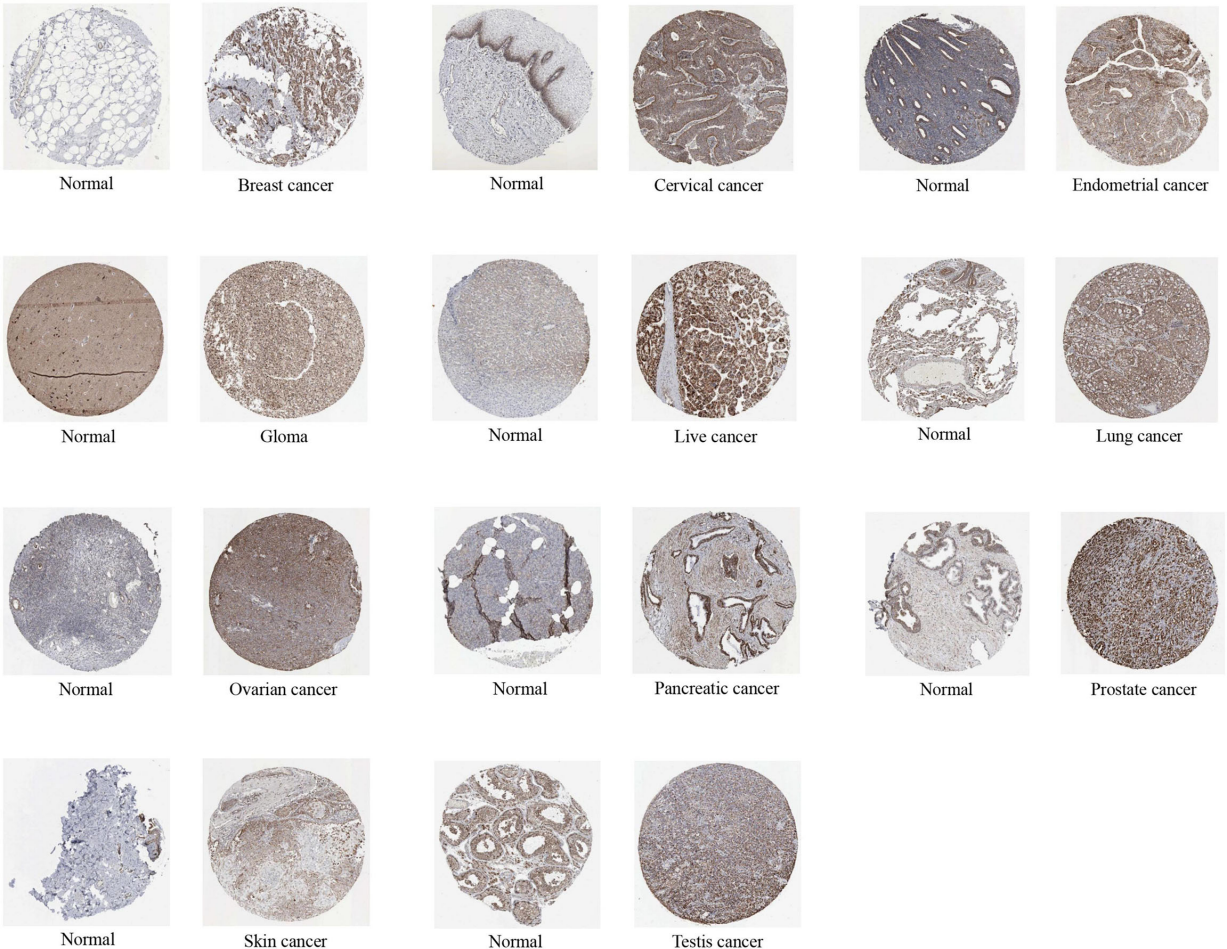
The protein expression of ACO2 in immunohistochemical images of normal (left) and tumor (right) groups.

In this study, we investigated the oncogenic function of ACO2 (mRNA: NM_001098.3, protein: NP_001089.1, **Figure S1**), which catalyzes the interconversion of citrate to isocitrate *via* cis-aconitate in the second step of the TCA cycle (**Figure 1**). On the basis of TCGA data, we compared ACO2 expression levels between cancer and paired normal samples across 33 cancer types. As shown in **Figure 2**, we found increased ACO2 expression compared with corresponding normal tissue in 16 tumor types, including breast invasive carcinoma (BRCA), cervical squamous cell carcinoma and endocervical adenocarcinoma (CESC), cholangiocarcinoma (CHOL), lymphoid neoplasm diffuse large B cell lymphoma (DLBC), esophageal carcinoma (ESCA), kidney chromophobe (KICH), brain lower grade glioma (LGG), liver hepatocellular carcinoma (LIHC), lung squamous cell carcinoma (LUSC), ovarian serous cystadenocarcinoma (OV), pancreatic adenocarcinoma (PAAD), prostate adenocarcinoma (PRAD), skin cutaneous melanoma (SKCM), testicular germ cell tumors (TGCT), thymoma (THYM), and uterine corpus endometrial carcinoma (UCEC) (p<0.05). Conversely, five tumor types showed decreased ACO2 expression: adrenocortical carcinoma (ACC), bladder urothelial carcinoma (BLCA), glioblastoma multiforme (GBM), kidney renal clear cell carcinoma (KIRC), and acute myeloid leukemia (LAML) (p<0.05). There were significant differences in ACO2 expression in most cancer types compared with corresponding normal tissues, indicating that ACO2 may play a potentially pivotal role in cancer diagnostics. Furthermore, for the 21 tumor types with differential ACO2 expression, we found available immunohistochemical images for ACO2 staining from the HPA database to assess ACO2 protein levels. These data showed that the trends of ACO2 expression were consistent at the gene and protein levels (**Figure 3**).

**Figure 4:**
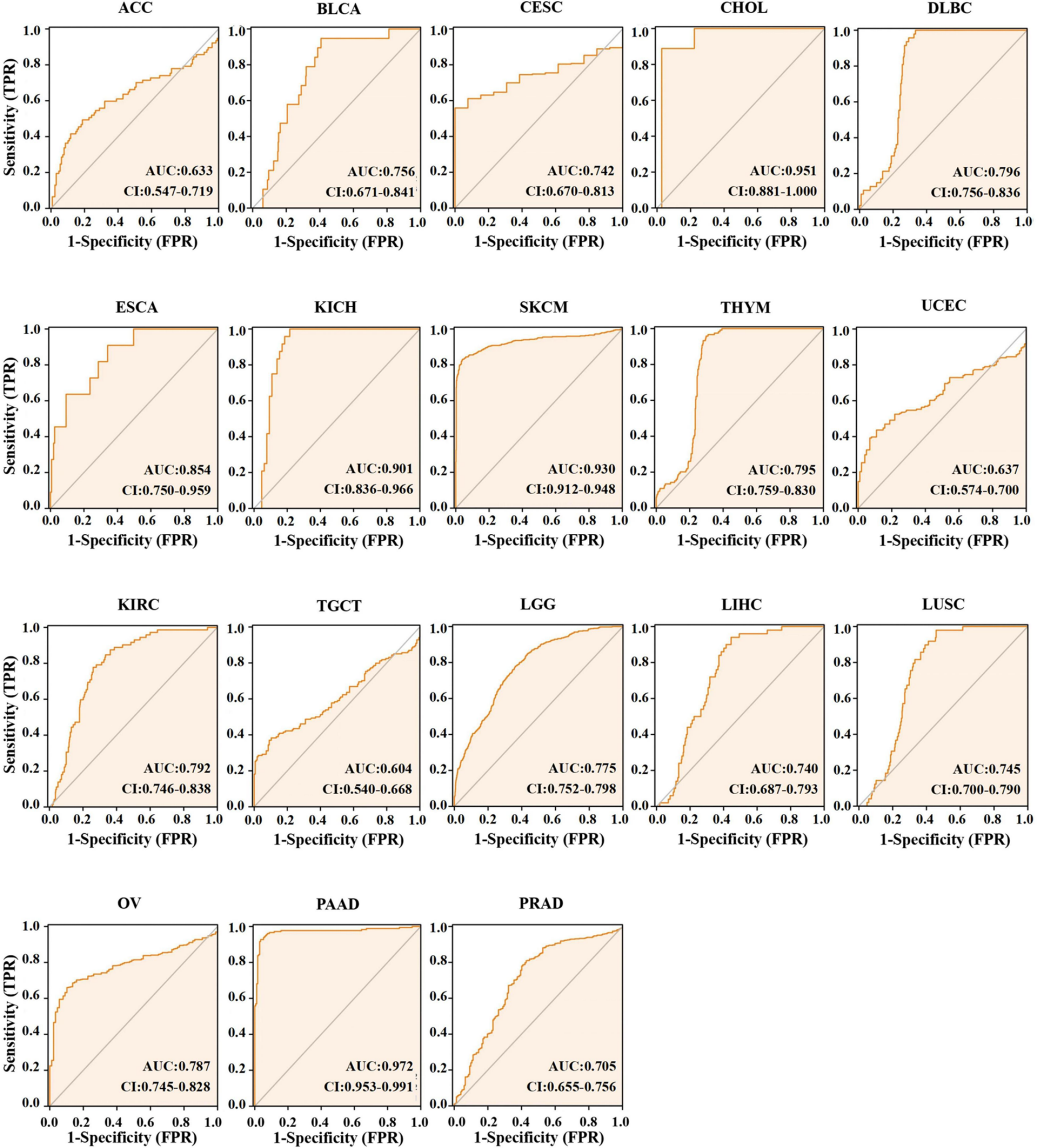
AUC of ROC curves verified the diagnosis performance of ACO2 in the TCGA and GTEx cohort.

Different AUC cut-offs are considered to indicate high diagnostic accuracy (AUC: 1.0–0.9), relative diagnostic accuracy (AUC: 0.9–0.7), and low diagnostic accuracy (AUC: 0.7–0.5). Our analysis of the diagnostic value of ACO2 was performed for the 21 types of cancers. As shown in **Figure 4**, the AUC of ROC analysis from the model had high diagnostic accuracy for four types of cancer, including CHOL, KICH, SKCM and PAAD, relative diagnostic accuracy for 11 types of cancer, and low diagnostic accuracy for three types of cancer, indicating that ACO2 may have important clinical value in the early diagnosis of these tumor types.

**Figure 5:**
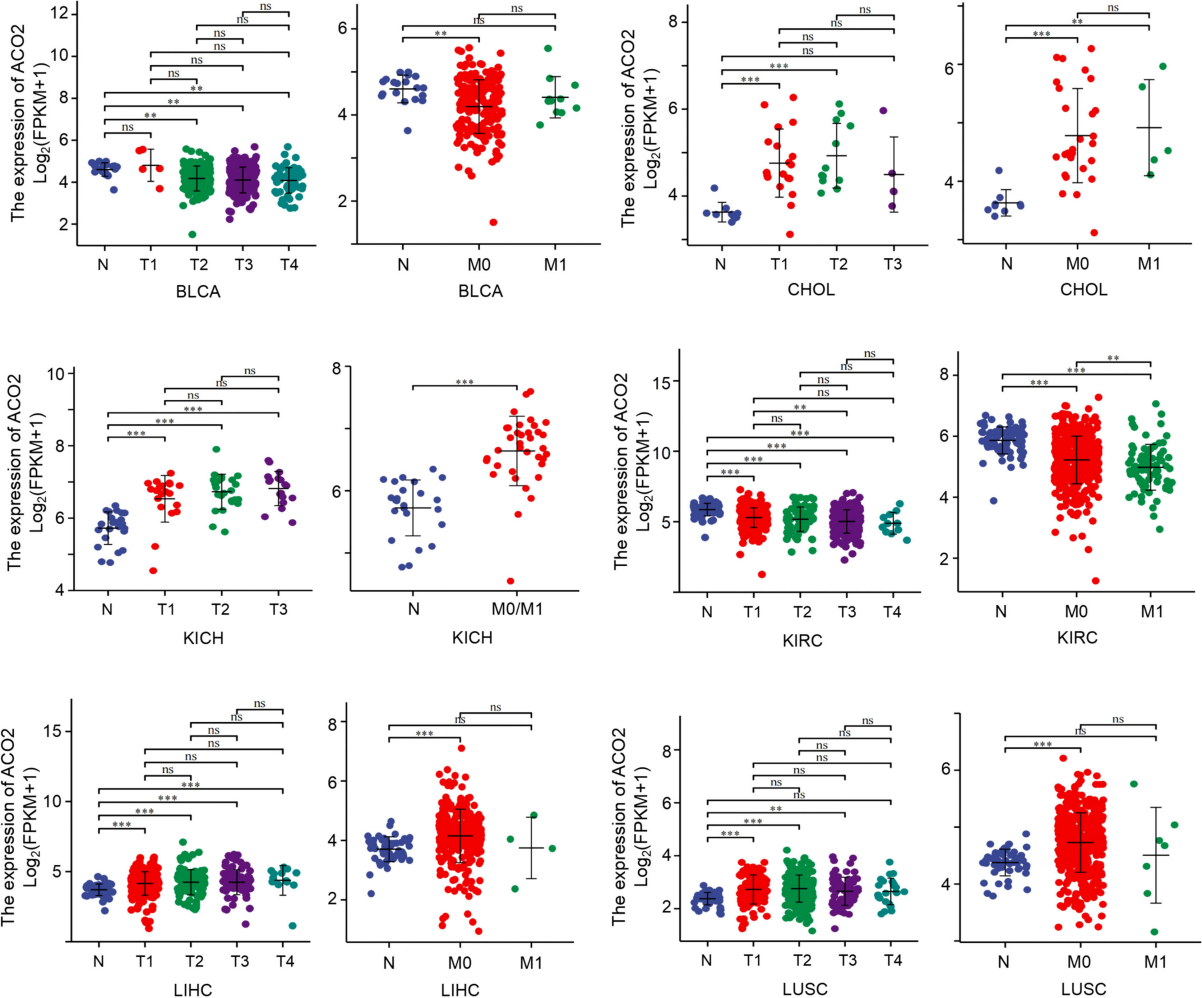
Association between ACO2 expression and tumor stage. **p < 0.01, ***p < 0.001. ns, statistically significant.

Next, among the 21 tumor types with differential ACO2 expression, we also investigated whether there were associations between stage and ACO2 levels. We discovered that four cancer types had significantly increased ACO2 expression in early tumor stages, including CHOL, KICH, LIHC, and LUSC, while ACO2 expression was decreased in early-stage BLCA and KIRC (**Figure 5**).

### Multifaceted prognostic value of ACO2 in cancers

**Figure 6:**
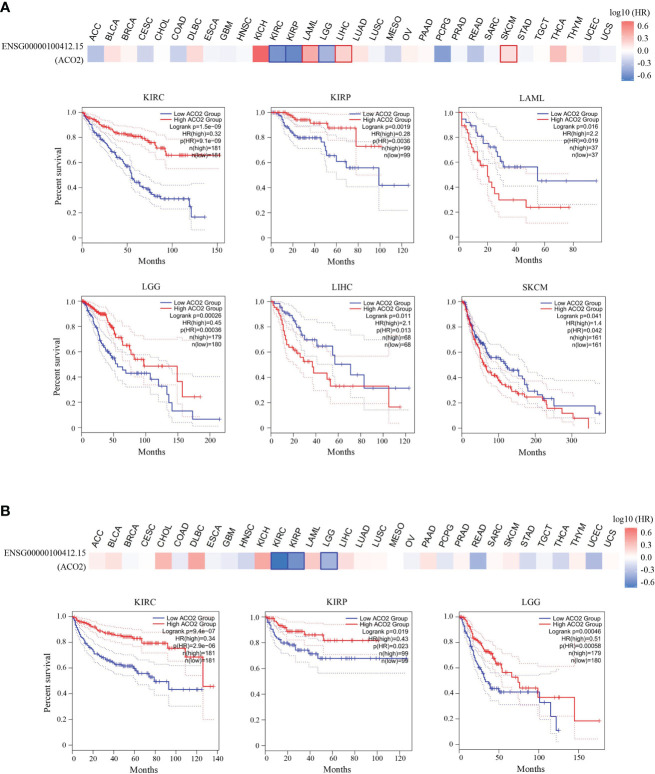
Correlation between ACO2 gene and the survival outcome of tumors. OS **(A)** and DFS **(B)** analyses of various tumors. The survival map and Kaplan-Meier curves with positive results are displayed.

With the aim of investigating associations between ACO2 expression and prognosis, we performed a survival association analysis using GEPIA2 for each cancer. According to ACO2 expression levels, we divided all cases into two groups, the high-expression and low-expression groups. As shown in **Figure 6A**, we found that ACO2 was associated with poor prognosis (OS) in LAML, LIHC, and SKCM (p<0.05). Meanwhile, for KIRC, kidney renal papillary cell carcinoma (KIRP), and LGG, high ACO2 expression was a favorable prognostic factor. Similarly, DFS analysis (**Figure 6B**) also showed a negative correlation between high ACO2 expression and prognosis for KIRC, KIRP, and LGG (p<0.05).

### Correlations between ACO2 and TMB/MSI

**Figure 7:**
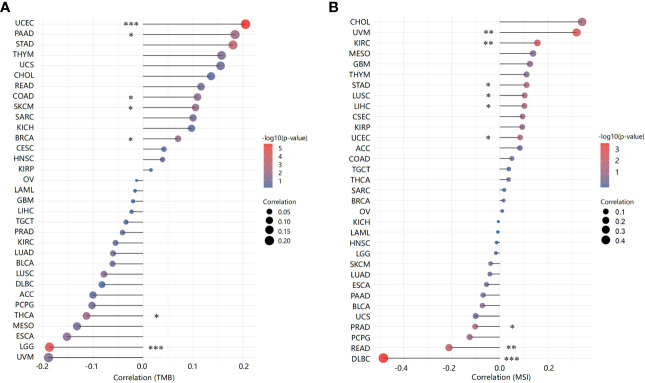
Correlation between ACO2 expression and tumor mutational burden and microsatellite instability. **(A)** Tumor mutational burden. **(B)** Microsatellite instability. *p < 0.05, **p < 0.01, ***p < 0.001.

Next, we analysed correlations between ACO2 expression and TMB/MSI across all tumors of TCGA. We observed a negative correlation between ACO2 expression and TMB for LGG (p<0.001) and thyroid carcinoma (THCA) (p<0.05) but found a positive correlation for UCEC (p<0.001), PAAD, colon adenocarcinoma (COAD), SKCM, and BRCA (p<0.01) (**Figure 7A**; **Supplement Table 1**). ACO2 expression was also positively correlated with MSI in uveal melanoma (UVM), KIRC (p<0.01), stomach adenocarcinoma (STAD), LUSC, and UCEC (p<0.05) but negatively correlated with MSI in DLBC (p<0.001), READ (p<0.01), and PRAD (p<0.05) (**Figure 7B**; **Supplement Table 2**). These results deserve more in-depth research.

### Relationship between ACO2 expression and tumor immune cell infiltration

Tumor-infiltrating immune cells, as prominent components of the tumor microenvironment, are closely associated with tumorigenesis and cancer progression and metastasis (33). Herein, the TIMER, EPIC, CIBERSORT, CIBERSORT-ABS, QUANTISEQ, and MCPCOUNTER algorithms were used to explore potential relationships between ACO2 expression and the level of infiltration of different immune cells, including CD8+ T cells, CD4+ T cells, TANs, TAMs, B cells, and NK cells, in diverse cancer types.

**Figure 8:**
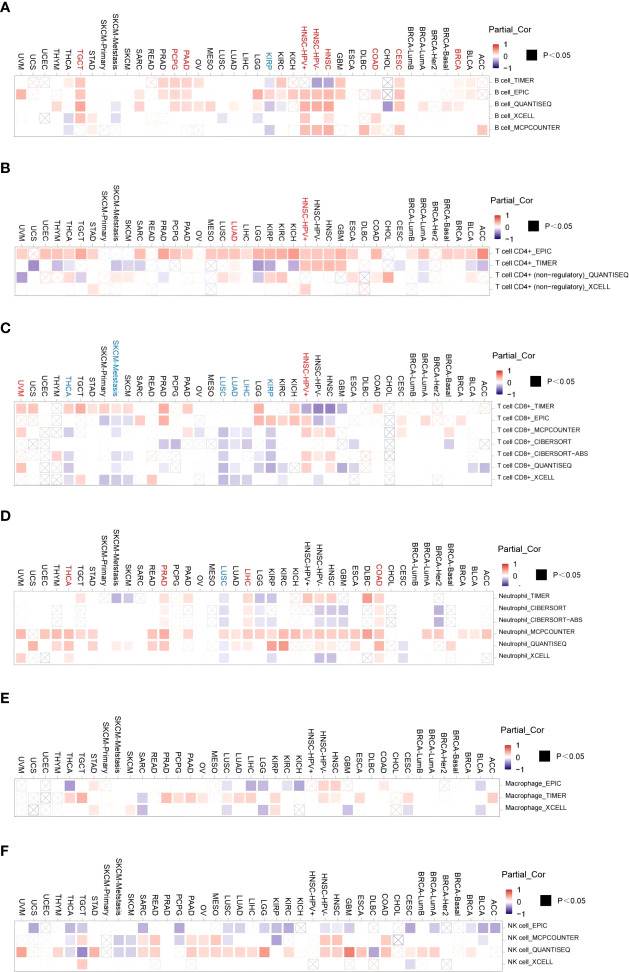
Correlation of ACO2 expression with immune infiltration level in diverse cancer types. Correlation analysis between the expression of ACO2 and immune infiltration of B cell **(A)**, CD4+T cell **(B)**, CD8+T cell **(C)**, tumor-associated neutrophil **(D)**, tumor-associated macrophage **(E)**, and NK cell **(F)** in different cancers.

**Figure 9:**
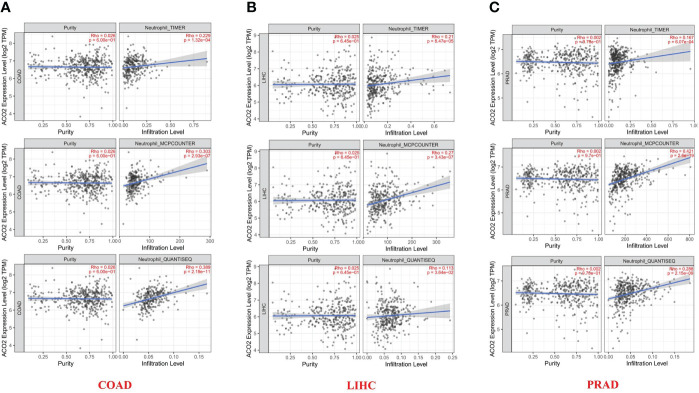
Relationship between infiltrates estimation value of tumor-associated neutrophil and ACO2 expression in COAD **(A)**, LIHC **(B)** and PRAD **(C)** using three algorithms.

After a series of analyses, we observed a significant statistical correlation between ACO2 expression and tumor immune cell infiltration, including B-cell infiltration in BRCA, COAD, and HNSC (**Figure 8A**), CD4+ cells in HNSC-HPV+ and LUAD (**Figure 8B**), CD8+ T cells in KIRP, LIHC, and LUAD (**Figure 8C**), and TANs in some cancers, especially COAD (**Figure 8D**). However, regarding the infiltration of TAMs (**Figure 8E**) and NK cells (**Figure 8F**), no correlations were found between ACO2 expression in various cancers. Taking COAD (**Figure 9A**), LIHC (**Figure 9B**) and PRAD (**Figure 9C**) as examples, the positive correlation between infiltrate estimation values for TANs and ACO2 expression was shown using three of all algorithms.

**Figure 10:**
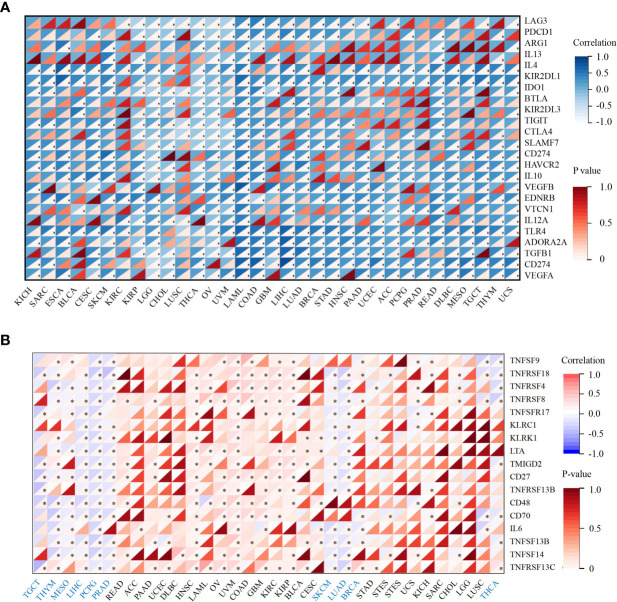
Correlation analysis between ACO2 expression and immune checkpoint and immunostimulators in corresponding tumors. **(A)** The relationship between ACO2 and immune checkpoint-associated genes. **(B)** The relationship betwwen ACO2 and immunostimulators. *p < 0.05.

Next, we performed correlation analyses between ACO2 expression and immune checkpoint-associated genes in corresponding tumors. The results showed that for most types of cancer, there was a significant correlation between ACO2 expression and the levels of immune checkpoint-associated genes (**Figure 10A**). In addition, our findings first revealed that ACO2 was positively correlated with multiple immunostimulators in some cancers, such as LIHC, PCPG, and PRAD (**Figure 10B**).

### Genetic alteration and methylation analysis of ACO2

**Figure 11:**
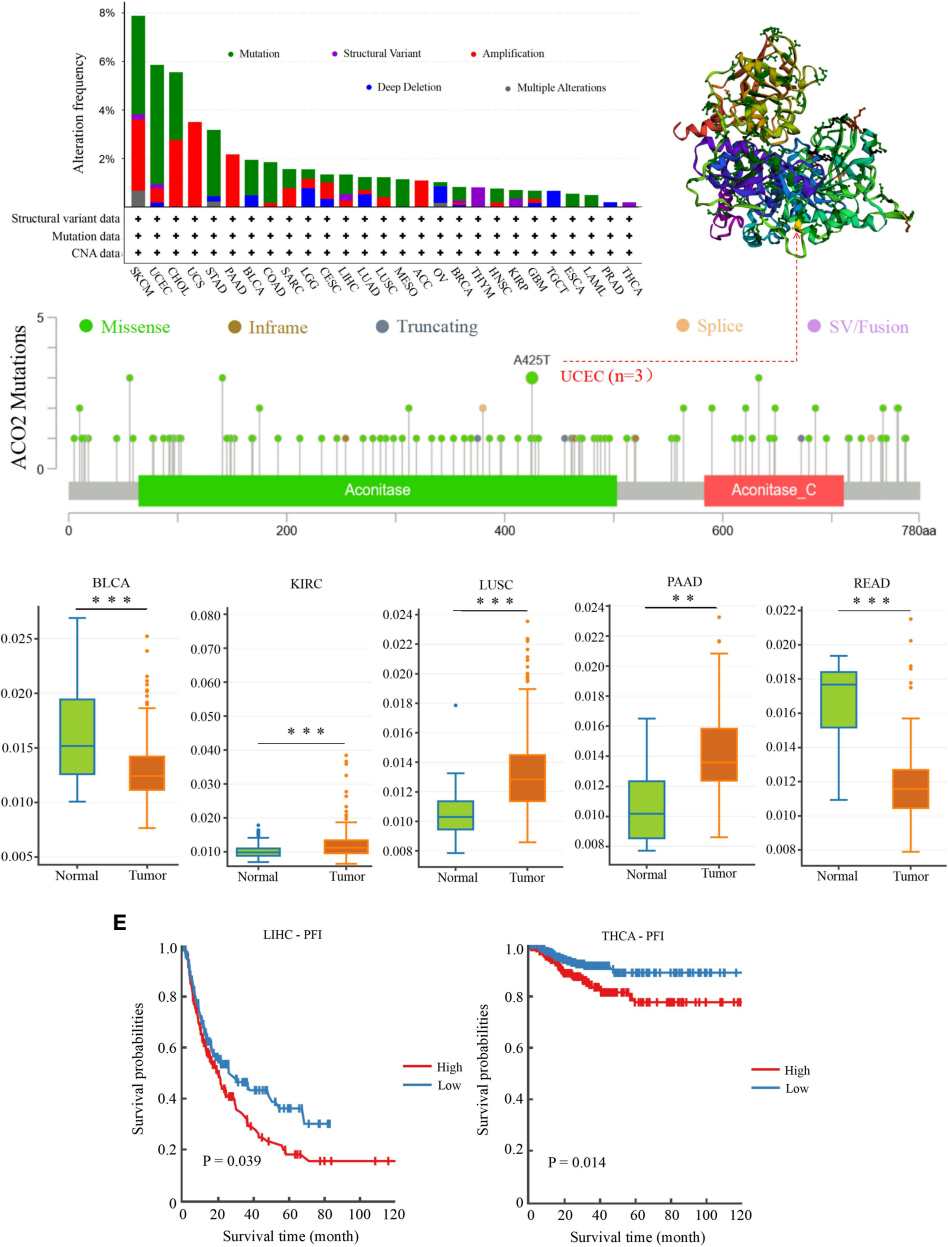
Genetic mutation and methylation analysis of ACO2 across various tumors. **(A)** The alteration frequency with mutation type. **(B)** The mutation site of ACO2 gene. **(C)** The mutation site with the highest alteration frequency (A425T) in the 3D structure of ACO2. **(D)** It shows higher methylation level of ACO2 in BLCA, KIRC, LUSC, PAAD, and READ. **(E)** The potential correlation between ACO2 methylation and the PEI of LIHC and THCA. **p < 0.01, ***p < 0.001.

Genetic alterations of ACO2 in various tumor types in TCGA datasets were investigated using cBioPortal. As shown in **Figure 11A**, we found that SKCM tumor samples had the highest frequency of ACO2 genetic alteration (close to 8%). Most of the genetic alterations occurring in SKCM tumor samples were copy number mutations, which was also the major type of genetic alteration in most TCGA tumor samples. In addition to SKCM, >4% of UCEC and CHOL samples showed genetic alterations of ACO2.

The types, sites, and case numbers of ACO2 genetic alterations are further presented in **Figure 11B**. We found that missense mutation of ACO2 was the main type of genetic alteration, with the A425T substitution in the Aconitate domain, which was detected in three UCEC cases and was the most common. Moreover, the A425T site can be observed in the 3D structure of the ACO2 protein in **Figure 11C**. We then further explored whether ACO2 genetic alterations were associated with clinical outcomes in various tumor types. However, regarding prognosis, there were no statistically significant differences for cases with or without ACO2 mutations.

We then used DNMIVD to investigate potential associations between ACO2 methylation and the pathogenesis of different tumors in TCGA. We found that the methylation levels of ACO2 were significantly increased in KIRC, LUSC, and PAAD (p<0.01) and decreased in BLCA and READ (**Figure 11D**; **Supplement Table 3**). Next, we explored the link between ACO2 methylation and tumor prognosis and found that high ACO2 methylation was associated with poor PFI compared with low methylation in LIHC and THCA (**Figure 11E**).

### Experimental validation of the expression and function of ACO2 in HCC

**Figure 12:**
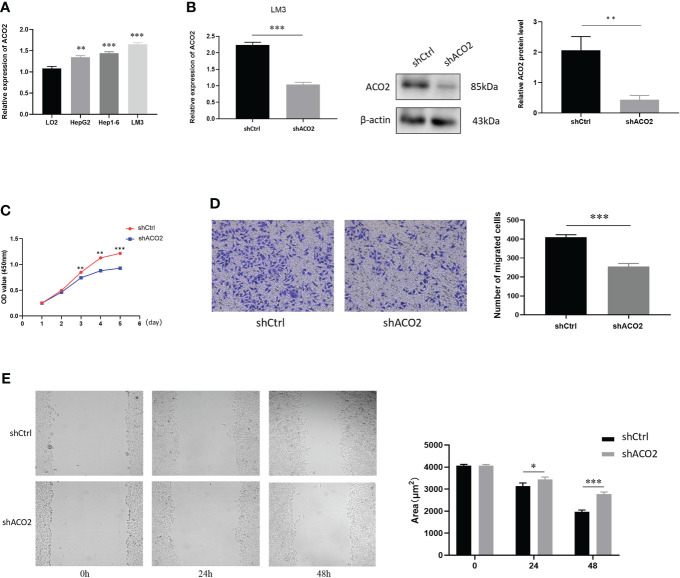
The expression of ACO2 in HCC cells and its effect on HCC proliferation and migration. **(A)** The expression level of ACO2 in normal hepatocyte and tumor cells. **(B)** knockdown of ACO2 gene in LM3 cell. **(C)** ACO2 promotes the migration of tumor cell. **(D, E)** ACO2 promotes the proliferation of tumor cell. *p< 0.05, **p < 0.01, ***p < 0.001.

According to our analysis of TCGA data, there were significant statistical associations in ACO2 expression according to tumor stage, prognosis, and diagnosis in HCC. Hence, we detected the expression of ACO2 in normal hepatocytes and HCC cells by qPCR and found that compared with LO2 cells, ACO2 expression was significantly increased in HepG2, LM3, and Hep1-6 cells (**Figure 12A**). To explore the effect of ACO2 on the progression of HCC, we knocked down ACO2 in LM3 cells by transecting a shRNA, the effects of which were verified by qPCR and western blot (**Figure 12B**). Next, we performed the CCK-8 assay and found that compared with the control group, the proliferation of ACO2-silenced HCC cells was significantly inhibited (**Figure 12C**). Moreover, wound-healing and Transwell migration assays were performed to evaluate the migration of HCC cells. HCC cells in the shACO2 group underwent a slower closing of scratch wounds than control cells (**Figure 12D**). Similarly, significantly increased numbers of siACO2 LM3 cells were observed on the outside membranes of Transwell chambers compared with the control group (**Figure 12E**).

### Enrichment analysis of ACO2-related partners

**Figure 13:**
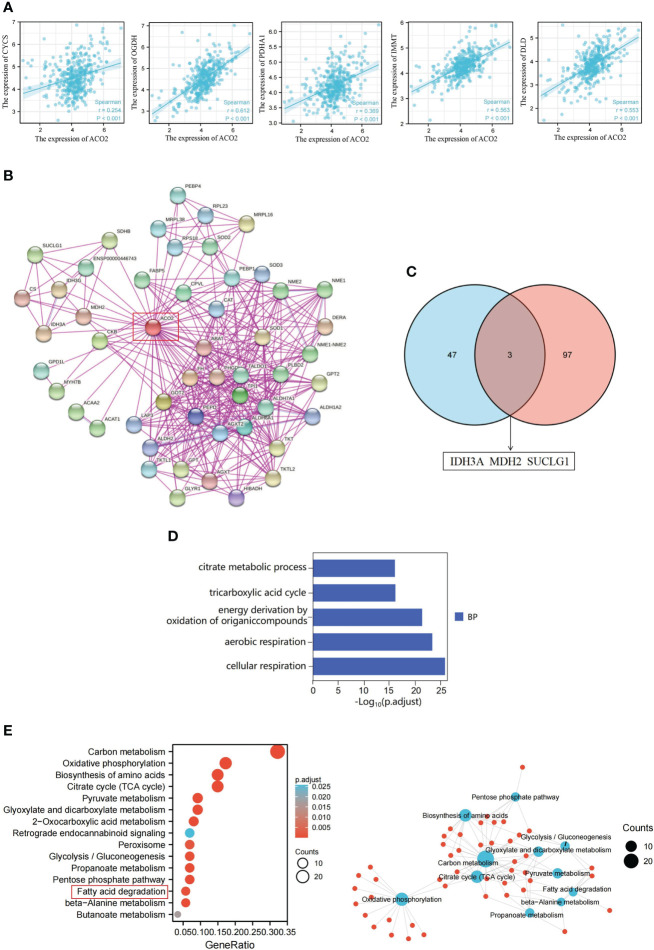
ACO2-related gene enrichment analysis in HCC. **(A)** Expression correlation between ACLY and CYCS, OGDH, PDHA1, IMMT and DLD. **(B)** ACO2-binding proteins. **(C)** The intersection of the ACO2-binding and correlated genes. **(D)** GO analysis. **(E)** KEGG pathway analysis.

To further investigate the molecular mechanism of the ACO2 gene in tumorigenesis, we attempted to screen ACO2-binding proteins and the genes correlated with ACO2 expression from a series of pathway enrichment analyses. First, we used the GEPIA2 tool to obtain the top 100 genes that correlated with ACO2 expression (**Supplement Table 4**). As shown in **Figure 13A**, taking the example of five genes with higher Pearson’s correlation coefficient, cytochrome C (CYCS) (R=0.254), dihydrolipoamide dehydrogenase (DLD) (R=0.553), inner membrane mitochondrial protein (IMMT) (R=0.563), oxoglutarate dehydrogenase (OGDH) (R=0.612), and pyruvate dehydrogenase E1 subunit alpha 1 (PDHA1) (R=0.369) (all p<0.01) were positively correlated with ACO2 expression. Then, using the STRING tool, we obtained a total of 50 ACO2-binding proteins, which were supported by experimental evidence. **Figure 13B** shows the interaction network of these proteins. An intersection analysis of the above two groups showed three common members, isocitrate dehydrogenase (NAD(+)) 3 catalytic subunit alpha (IDH3A), malate dehydrogenase 2 (MDH2), and succinate-CoA ligase GDP/ADP-forming subunit alpha (SUCLG1) (**Figure 13C**).

Next, we combined the two datasets to perform KEGG and GO enrichment analyses. The GO enrichment analysis demonstrated that ACO2 may be involved in the process of cellular energy metabolism and play an important role in coenzyme binding, DNA binding, and electron transfer activity during LIHC pathogenesis (**Figure 13D**). KEGG data suggested that ACO2 regulates tumor progression by affecting molecular pathways involved in cellular energy metabolism and metabolite changes (**Figure 13E**). Significantly, we found that the fatty acid metabolic pathway may play an important role in the pathogenesis of LIHC. Next, the enrichment network analysis of ACO2-related proteins and genes was performed, from which we observed the interassociation of mechanistic molecular pathways, which may be useful for future research.

### Enrichment analysis of ACO2-related genes in the scRNA-seq dataset

**Figure 14:**
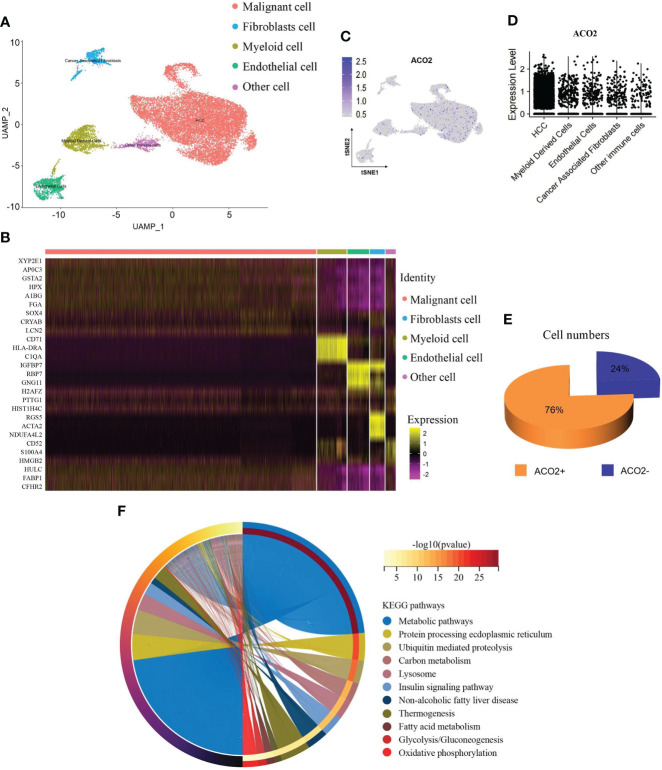
ScRNA-seq analysis reveals the expression and function of ACO2 in HCC. **(A)** UMAP plots show cells isolated from HCC, colored by the main cell groups. **(B)** Heatmap showing the expression of marker genes in the indicated cell types. **(C, D)** Dot plot shows ACO2 expression in each cell group. **(E)** The pie graph shows the proportion of ACO2^+^/ACO2^-^ cells of malignant cells. **(F)** KEGG pathway analysis.

We used the scRNA-seq dataset by Shen et al. (30), in which close to 20000 cells passed standard quality control and were retained for subsequent analysis. We mainly clustered all cells into five subsets (**Figure 14A**) and annotated the cell population with previously published marker genes, including malignant cells (MDK, GPC3), fibroblast cells (ACTA2, MYL9), myeloid cells (CD74), endothelial cells (CDH5, VWF) and other cells (**Figure 14B** and **Figure S2**). By locating the expression of ACO2, we found that ACO2 was mainly expressed in malignant cells (**Figure 14C** and **Figure 14D**). In addition, we found that the proportion of ACO2+ cells in HCC was obviously higher than that in ACO2- cells (**Figure 14E**).

To examine the potential role of ACO2+ malignant cells in HCC, we separated malignant cells into two subgroups according to the expression of ACO2 to screen differentially expressed genes (**Supplement Table 5**) and analysed the differential KEGG pathways (**Figure 14F**). We found that ACO2+ malignant cells were mainly involved in metabolic pathways. Similarly, fatty acid metabolism was also found, which further suggests that ACO2 may regulate tumor progression by affecting fatty acid metabolism.

### Changes in lipid metabolite patterns in HCC cells following ACO2 knockdown

**Figure 15:**
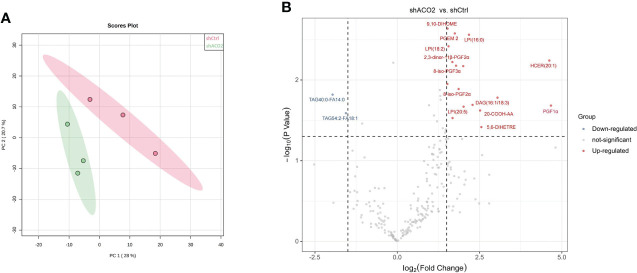
The results of cell Lipidomics. **(A)** The principal component analysis. **(B)** The colcano plot. Vocano plot indicates the distribution of differentially expressed lipid metabolites in LM3 with or without knockdown of ACO2 (n=3, Down: p < 0.05 and log_2_FC < 1.5; Up: p < 0.05 and log_2_FC >1.5).

Then, to determine the effect of ACO2 loss on lipid metabolism, we analysed the lipid profiles of LM3 cells with or without ACO2 knockdown using liquid chromatography−mass spectrometry (LC−MS). Principal component analysis (PCA) showed significant between-group differences (**Figure 15A**). The results revealed that shACO2 cells had a specific group of metabolites that was different from control cells (**Supplemental Table 6**). Moreover, a differential analysis showed a total of 19 significantly altered lipids after knockdown of ACO2 (log2fold change > 1.5 or < -1.5, P < 0.05, **Supplemental Table 7**), including 2 reduced metabolites, such as, and 17 increased metabolites, such hexosyl-ceramide (HCER (20:1)), prostaglandin F1 alpha (PGF1α), and 20-carboxy arachidonic acid (20-COOH AA) (**Figure 15B**).

At present, metabolites traditionally associated with bioenergetics or biosynthesis have been implicated in immunity and malignancy (34, 35). In subsequent experiments, we will further perform cell- or animal-based experiments to verify the function of ACO2 in HCC and determine whether ACO2 can regulate HCC progression by affecting changes in downstream metabolites.

## Discussion

To date, cancer-related research has always been a research focus in the current medical domain. Accumulating evidence has suggested a link between abnormalities in TCA cycle enzymes and tumorigenesis. Genetic defects, including IDH, SDH, and FH, have been investigated for metabolic enzyme-mediated oncogenesis (6–8). Mitochondrial ACO2 catalyzes the interconversion of citrate to isocitrate in the second step of the TCA cycle. Six-carbon citrate is exported across the mitochondrial membrane to the cytosol and further converted into oxaloacetate and two-carbon acetyl-CoA, which is the precursor for fatty acid synthesis. As such, ACO2 is an essential enzyme that bridges the TCA cycle and lipid metabolism. However, few studies on ACO2 and tumor development have been reported. Thus, in this study, we systemically characterized ACO2 in a variety of tumor types by analysing features such as gene expression, genetic alteration, methylation, and immune infiltration.

To include more samples, 33 cancer-related datasets from TCGA and GTEx were used to explore biomarkers suitable for cancer diagnosis through gene expression difference analysis. Except for cancers with no normal tissue data, our results detected significant differences in ACO2 expression between tumors and normal tissues of 21 forms of cancer, and analysis of available immunohistochemical images confirmed this tendency at the protein level. Among them, ACO2 was upregulated in most cancers, such as BRCA, CESC, CHOL, and DLBC, and five forms of cancer showed downregulation between tumor and nontumor tissues (ACC, BLCA, GBM, KIRC and LAML). Unfortunately, in UVM and MESO, this analysis was unsuccessful due to a lack of normal control samples. With the accumulation of datasets, this will be worthy of further exploration in the future. Xin You et al. (13) showed that ACO2 expression was decreased in colorectal cancer, and we explored the expression of ACO2 in rectal cancer and colon cancer. No significant difference was found, both of which cannot be compared effectively. Peng Wang et al. (36) showed that ACO2 expression was decreased in gastric cancer, which was different from our current results, possibly because fewer normal samples (n=30) were included in previous research. In addition, regarding breast cancer, our findings challenge those of previous research (12), which extracted and assessed ACO2 expression by the Gene Expression Omnibus database (GSE15852 and GSE294318) and indicated that ACO2 was downregulated in tumor tissues. However, the information about GSE294318 was not found in GEO datasets, and we could not evaluate the validity of the data. Overall, these findings confirm that ACO2 expression is upregulated in a variety of cancers, suggesting that the prospect of ACO2 in cancer diagnosis is worth looking forwards to.

Currently, early cancer detection is of great clinical significance to push back the frontier of early cancer detection. The AUC is a standard used to measure the quality of the classification model. Gonzalez-Ericsson PI et al. (37) tested the ability of MHC-II expression on tumor cells, to predict immunotherapy-specific benefit in the neoadjuvant breast cancer setting and revealed that quantitative assessment of MHC-II on tumor cells was predictive of durvalumab + NAC and pembrolizumab + NAC (ROC AUC, 0.71; P = 0.01 and AUC, 0.73; P = 0.001, respectively), but not NAC alone (AUC, 0.5; P = 0.99). Weidhaas J et al. (38) tested the hypothesis that germline microRNA pathway functional variants, known to predict altered systemic stress responses to cancer therapies, would predict irAEs in patients across cancer types, and found that a biomarker panel was identified that predicts toxicity with 80% accuracy (F1 = 0.76, area under the curve (AUC)=0.82) in the melanoma training cohort and 77.6% accuracy (F1 = 0.621, AUC=0.778) in the pan-cancer validation cohort. For the 21 types of cancers with differential expression of ACO2, the AUC of the ROC curve revealed the superior performance of ACO2 in the diagnosis of 18 of them, especially CHOL, KICH and PAAD. Then, we explored the differential expression of ACO2 in samples marked with staging information. For the 21 types of cancers, there were 17 cancers in which staging and normal control samples were collected. Our analysis showed a similar trend of ACO2 expression only in terms of tumor stage in 6 of the 17 cancers, which may be related to the small sample sizes of different tumor stages. In brief, the results may provide useful insight for the early diagnosis of some tumors.

To investigate associations between ACO2 expression and prognosis, a survival analysis was performed using Kaplan-Meier survival curves for each type of cancer, including OS and DFS. By combining these results, we found that high ACO2 expression was linked to poor prognosis (OS) in LAML, LIHC, and SKCM, while for KIRC, KIRP, and LGG, high ACO2 expression was a favorable prognostic factor according to OS and DFS.

Increasing evidence indicates a link between genomic mutations and methylation status and tumor progression (39, 40). For instance, studies have found that PRC2 inactivation increases sensitivity to genetic or small molecule inhibition of DNA methyltransferase 1 (DNMT1), resulting in enhanced cytotoxicity and antitumour responses (41). In this study, from the results interpreted in cBioPortal, we found that mutations in ACO2 were most common in SKCM, followed by UCEC, CHOL, UCS, STAD and PAAD, which suggests that we should pay attention to the relationship between ACO2 gene mutation and digestive system and female reproductive system tumors. Changes in DNA methylation in cancer have been heralded as promising targets for the development of powerful prognostic and predictive biomarkers (42). In addition, DNA methylation profiling is an additional emerging tool that will serve as an adjunct to increase the accuracy of pathological diagnosis (43). In our study, an elevated methylation level of ACO2 was found in LUSC and PAAD, in which ACO2 expression was also significantly increased. For LUSC and PAAD, more research is needed to determine whether ACO2 can be used as an auxiliary diagnostic biomarker.

Recent studies have shown that tumors with high MSI and TMB show a better response to immunotherapy (44). Our results showed that the expression of ACO2 was closely related to MSI and TMB in some tumors. ACO2 expression correlated with the expression of multiple immune checkpoint-associated genes, which are important drug targets of immunotherapy. In addition, we also analysed the correlation between ACO2 expression and immunostimulators and found that there was a clear negative correlation between ACO2 expression and multiple immunostimulators in some tumors, especially LIHC, PCPG, PRAD, SKCM and LUAD. Therefore, ACO2 may be a potential predictive biomarker of immunotherapeutic responses for some tumors.

Growing evidence suggests that innate immune cells (macrophages, neutrophils, dendritic cells, innate lymphoid cells, myeloid-derived suppressor cells, and natural killer cells) as well as adaptive immune cells (T cells and B cells) contribute to tumor progression when present in the tumor microenvironment (45). By investigating correlations between ACO2 expression and levels of tumor immune cell infiltration, we first found that ACO2 expression was positively correlated with the level of infiltration of TANs in COAD, LIHC, and PRAD and was negatively correlated with M1 macrophages in LUSC. TANs can participate in tumour-promoting inflammation by driving angiogenesis, extracellular matrix remodelling, metastasis and immunosuppression. Conversely, neutrophils can also mediate antitumour responses by direct killing of tumour cells and by participating in cellular networks that mediate antitumour resistance. Neutrophil diversity and plasticity underlie the dual potential of TANs in the tumour microenvironment (46). At present, there are no relevant studies on ACO2 and immune cells in tumors. Whether ACO2 promoted the infiltration of TANs into some tumors and affected tumor progression is worthy of further investigation.

Furthermore, *in vitro* experiments with HCC showed that ACO2 expression was significantly increased in HCC cells compared with hepatic cells, which was consistent with the result of Yilin Pang et al. (47). Our experimental results first revealed that ACO2 acted as an oncogene and promoted the growth, migration, and invasiveness of HCC cells. Gene co-expression is a kind of analysis method that uses a large number of gene expression data to construct correlations between genes and explore gene functions. We screen the top 100 ACO2-correlated targeting genes and 50 ACO2-binding proteins detected by anti tag coimmunoprecipitation assay to further investigate the tumor-promoting mechanism of ACO2 in HCC. An intersection analysis of the above two groups showed three common members, including IDH3A, MDH2, and SUCLG1, which may play an important role in tumor-promoting effect of ACO2. Then, enrichment analysis of ACO2-related genes using two approaches all revealed the significance of fatty acid metabolism. Significantly, previous studies have found that ACO2 could affect tumor development by regulating lipid metabolism in colorectal cancer and prostate cancer (11, 13). Thus, we further performed lipidomics using cells with or without ACO2 knockdown and screened 19 markedly changed metabolites. In future work, we will investigate whether ACO2 could promote the development of HCC by regulating the production of downstream metabolites.

## Conclusion

In summary, through a pan-cancer analysis, we found that ACO2 was widely differentially expressed between tumor and normal tissues and revealed a correlation between ACO2 expression and clinical prognosis in some tumor types.

ACO2 gene alterations, including mutations, duplications, and amplifications, were identified in a wide variety of cancer types. ACO2 expression showed a significant association with the infiltration of some immune cells into the tumor microenvironment and immunostimulators in multiple cancers. Therefore, for some tumors, including HCC, ACO2 may be a potential diagnostic biomarker and predictive biomarker of immunotherapeutic responses.

## Data availability statement

The original contributions presented in the study are included in the article/**Supplementary Material**. Further inquiries can be directed to the corresponding authors.

## Author contributions

HeC contributed to the conception and design of the study. ZW and WZ performed the experiments and drafted the manuscript. ZW, WZ, ZC, SW and HaC collected and analysed the data. MC and HeC revised the manuscript. All authors contributed to the article and approved the submitted version.

## Funding

This study was supported by the School Foundation of Anhui Medical University (2019XKJ182).

## Acknowledgments

We thank James P. Mahaffey, PhD, from Liwen Bianji (Edanz) (www.liwenbianji.cn) for editing the English text of a draft of this manuscript.

## Conflict of interest

The authors declare that the research was conducted in the absence of any commercial or financial relationships that could be construed as a potential conflict of interest.

## Publisher’s note

All claims expressed in this article are solely those of the authors and do not necessarily represent those of their affiliated organizations, or those of the publisher, the editors and the reviewers. Any product that may be evaluated in this article, or claim that may be made by its manufacturer, is not guaranteed or endorsed by the publisher.
