# Supplementary material for: Pan-Cancer analysis shows that ACO2 is a potential prognostic and immunotherapeutic biomarker for multiple cancer types including hepatocellular carcinoma
Source: Front Oncol. 2022 Nov 30;12:1055376. doi: 10.3389/fonc.2022.1055376 (PMC9748622; doi:10.3389/fonc.2022.1055376)
Supplement: Supplementary file 1 [file DataSheet_1.zip › Original data/R package-ggplot2.docx]

library(tidyverse)

library(ggplot2)

library(reshape2)

library(car)

library(rstatix)

set.seed(100)

data <- data.frame(x = rnorm(100, 2, 1), y = rnorm(100, 1, 1))

data2 <- melt(data)

data3 <- lapply(data, function(x) get_summary_stats(data.frame(x)))

data3

# $x

# # A tibble: 1 x 13

# variable n min max median q1 q3 iqr mad mean sd se ci

# <chr> <dbl> <dbl> <dbl> <dbl> <dbl> <dbl> <dbl> <dbl> <dbl> <dbl> <dbl> <dbl>

# 1 x 100 -0.272 4.58 1.94 1.39 2.66 1.26 0.974 2.00 1.02 0.102 0.203

#

# $y

# # A tibble: 1 x 13

# variable n min max median q1 q3 iqr mad mean sd se ci

# <chr> <dbl> <dbl> <dbl> <dbl> <dbl> <dbl> <dbl> <dbl> <dbl> <dbl> <dbl> <dbl>

# 1 x 100 -1.14 3.17 0.927 0.568 1.45 0.878 0.648 1.01 0.796 0.08 0.158

data3 <- rbind(data3[[1]], data3[[2]])

data3[1] <- c("x", "y")

## Shapiro-Wilk normality test

lapply(data, function(x) shapiro.test(x))

# $x

#

# Shapiro-Wilk normality test

#

# data: x

# W = 0.98836, p-value = 0.535

#

#

# $y

#

# Shapiro-Wilk normality test

#

# data: x

# W = 0.98532, p-value = 0.3348

## Levene's Test

leveneTest(value~variable, data = data2)

# Levene's Test for Homogeneity of Variance (center = median)

# Df F value Pr(>F)

# group 1 4.4476 0.03621 *

# 198

# ---

# Signif. codes: 0 â€˜***â€™ 0.001 â€˜**â€™ 0.01 â€˜*â€™ 0.05 â€˜.â€™ 0.1 â€˜ â€™ 1

t.test(value~variable, data = data2, var.equal = T)

# Two Sample t-test

#

# data: value by variable

# t = 7.6613, df = 198, p-value = 8.012e-13

# alternative hypothesis: true difference in means is not equal to 0

# 95 percent confidence interval:

# 0.7364913 1.2470521

# sample estimates:

# mean in group x mean in group y

# 2.002913 1.011141

t.test(value~variable, data = data2, var.equal = F)

# Welch Two Sample t-test

#

# data: value by variable

# t = 7.6613, df = 186.92, p-value = 9.657e-13

# alternative hypothesis: true difference in means is not equal to 0

# 95 percent confidence interval:

# 0.7363983 1.2471452

# sample estimates:

# mean in group x mean in group y

# 2.002913 1.011141

wilcox.test(value~variable, data = data2)

# Wilcoxon rank sum test with continuity correction

#

# data: value by variable

# W = 7844, p-value = 3.711e-12

# alternative hypothesis: true location shift is not equal to 0

summary(aov(value~variable, data = data2))

# Df Sum Sq Mean Sq F value Pr(>F)

# variable 1 49.18 49.18 58.7 8.01e-13 ***

# Residuals 198 165.90 0.84

# ---

# Signif. codes: 0 â€˜***â€™ 0.001 â€˜**â€™ 0.01 â€˜*â€™ 0.05 â€˜.â€™ 0.1 â€˜ â€™ 1

ggplot(data2, aes(x = variable, y = value, color = variable, fill = variable)) +

geom_violin(alpha = 0.2) +

theme_bw()

ggplot(data2, aes(x = variable, y = value, color = variable, fill = variable)) +

geom_violin(alpha = 0.2) +

geom_point(position = position_jitter(0.3)) +

theme_bw()

ggplot(data2, aes(x = variable, y = value, color = variable, fill = variable)) +

geom_boxplot(alpha = 0.2) +

geom_point(position = position_jitter(0.3)) +

theme_bw()

ggplot(data2, aes(x = variable, y = value, color = variable, fill = variable)) +

geom_violin(alpha = 0.1) +

geom_boxplot(alpha = 0.1) +

geom_point(position = position_jitter(0.3)) +

theme_bw()

ggplot() +

geom_violin(data = data2, aes(x = variable, y = value, color = variable, fill = variable), alpha = 0.1) +

geom_errorbar(data = data3, aes(x = variable, ymin=mean-sd, ymax=mean+sd), width = 0.2)
