# Supplementary figures and images for: Pan-Cancer analysis shows that ACO2 is a potential prognostic and immunotherapeutic biomarker for multiple cancer types including hepatocellular carcinoma
Source: Front Oncol. 2022 Nov 30;12:1055376. doi: 10.3389/fonc.2022.1055376 (PMC9748622; doi:10.3389/fonc.2022.1055376)

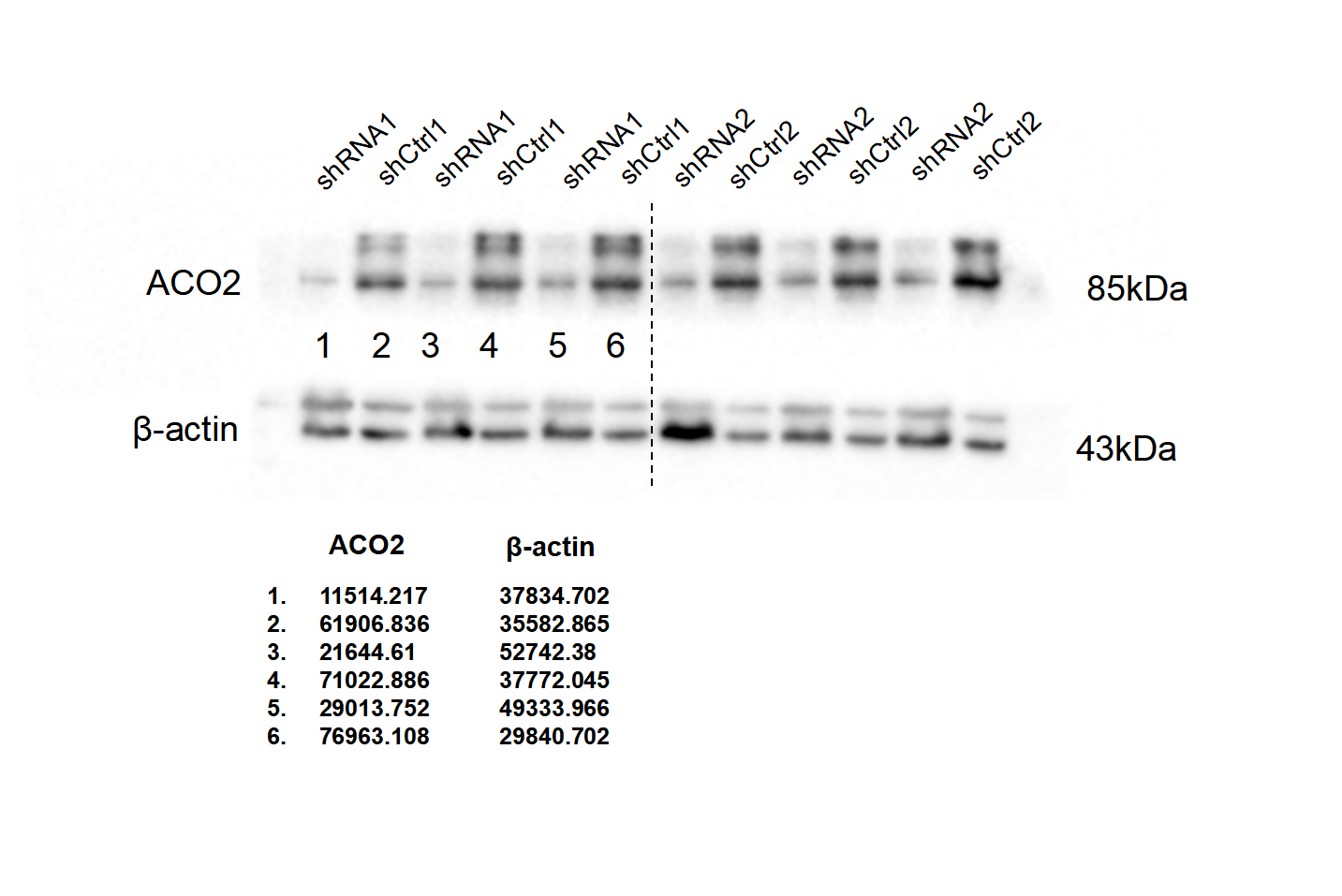

Supplement: Supplementary file 1 [file DataSheet_1.zip › Original data/Figure data/Figure 12B. WB.tif]
