# Supplementary material for: Pan-Cancer analysis shows that ACO2 is a potential prognostic and immunotherapeutic biomarker for multiple cancer types including hepatocellular carcinoma
Source: Front Oncol. 2022 Nov 30;12:1055376. doi: 10.3389/fonc.2022.1055376 (PMC9748622; doi:10.3389/fonc.2022.1055376)
Supplement: Supplementary file 1 [file DataSheet_1.zip › Original data/R Package-Seurat.doc]

\###

library(Seurat)

library(ggplot2)

library(cowplot)

library(Matrix)

library(dplyr)

library(stringr)

\###

Sample1 <- Read10X("./a")

Sample2 <- Read10X("./b")

Sample3 <- Read10X("./c")

write.csv(Sample1,file="./Sample1.csv")

write.csv(Sample2,file="./Sample2.csv")

write.csv(Sample3,file="./Sample3.csv")

path<-"data"

files <- list.files(path = path,pattern = 'csv$')

fullpath<-paste(path,files,sep="/")

names(fullpath) <- lapply(files,function(x) {str_extract(x, '[\\w\\d]+')})

rawdata <- lapply(fullpath, function(x) {

cells.table <- read.table(x, sep = ",",check.names = F, header = TRUE, row.names = 1)

})

\###

obj.list <- lapply(names(rawdata), function(x) {

CreateSeuratObject(counts = (rawdata[[x]]),

project = x,

min.cells = 10,

min.features = 200)})

names(obj.list) <- names(rawdata)

obj.list

\###

obj.list$Sample1@meta.data$group <- 'region a'

obj.list$Sample2@meta.data$group <- 'region b'

obj.list$Sample3@meta.data$group <- 'region c'

head(obj.list$Sample1@meta.data)

HB_all <- c("HBA1","HBA2","HBB","HBD","HBE1","HBG1","HBG2","HBM","HBQ1","HBZ")

for (x in names(obj.list)){

obj.list[[x]][["percent.MT"]] <- PercentageFeatureSet(obj.list[[x]], pattern = "^MT-")

obj.list[[x]][["percent.Ribo"]] <- PercentageFeatureSet(obj.list[[x]], pattern = "^RP[SL]")

HB_genes <- intersect(HB_all, rownames(obj.list[[x]]))

obj.list[[x]][["percent.HB"]] <- PercentageFeatureSet(obj.list[[x]], features = HB_genes)

}

\###

qc_feature <- c("nFeature_RNA", "nCount_RNA", "percent.HB", "percent.MT", "percent.Ribo")

for(sample in names(obj.list)){

pdf(file=paste0("1_",sample,"After_quality_control.pdf"),width = 15,height=7)

print(VlnPlot(obj.list[[sample]], features = qc_feature, ncol = 5, pt.size = 0.5))

dev.off()

}

\###

for(sample in names(obj.list)){

pdf(file=paste0("1_",sample,"After_feature_relationship.pdf"),width = 12,height=10)

p1 <- FeatureScatter(obj.list[[sample]], feature1 = "nCount_RNA", feature2 = "nFeature_RNA")

p2 <- FeatureScatter(obj.list[[sample]], feature1 = "nCount_RNA", feature2 = "percent.HB")

p3 <- FeatureScatter(obj.list[[sample]], feature1 = "nCount_RNA", feature2 = "percent.MT")

p4 <- FeatureScatter(obj.list[[sample]], feature1 = "nCount_RNA", feature2 = "percent.Ribo")

print(p1 + p2 + p3 + p4)

dev.off()

}

\###

obj.list <- lapply(obj.list, function(x) {

subset(x, subset = nCount_RNA < 40000 &

percent.MT < 25)})

obj.list

\###

obj.list <- lapply(obj.list,function(x) {

NormalizeData(x)

})

obj.list <- lapply(obj.list, function(x) {

FindVariableFeatures(x, selection.method = "vst", nfeatures = 2000)

})

obj.list

memory.limit(60000)

\###

obj.anchors <- FindIntegrationAnchors(object.list = obj.list, dims = 1:20)

obj.combined <- IntegrateData(anchorset = obj.anchors, dims = 1:20)

DefaultAssay(obj.combined) <- "integrated"

\###

all.genes <- rownames(obj.combined[["RNA"]]@data)

length(all.genes)

obj.combined <- ScaleData(obj.combined, features = all.genes)

\###

obj.combined <- RunPCA(obj.combined, npcs = 30, verbose = F)

obj.combined <- RunUMAP(obj.combined, reduction = "pca", dims = 1:20)

obj.combined <- FindNeighbors(obj.combined, reduction = "pca", dims = 1:20)

obj.combined <- FindClusters(obj.combined, resolution = 0.2)

\###

pdf('0.2_UMAP_multi_samples_combined.pdf',width = 11,height = 6)

p1 <- DimPlot(obj.combined, reduction = "umap", group.by = "group")

p2 <- DimPlot(obj.combined, reduction = "umap", label = TRUE)

p1 + p2

dev.off()

\###

pdf('0.2_UMAP_multi_samples_split.pdf',width = 10,height = 6)

DimPlot(obj.combined, reduction = "umap", split.by = "group",label = TRUE)

dev.off()

\###

sample.markers <- FindAllMarkers(obj.combined, only.pos = TRUE, min.pct = 0.25, logfc.threshold = 0.25)

top10 <- sample.markers %>% group_by(cluster) %>% top_n(n = 10, wt = avg_log2FC)

write.csv(file="4_top10_Cell_Markers.csv",top10,row.names=F)

\###

top10_table=unstack(top10, gene ~ cluster)

names(top10_table)=gsub("X","cluster",names(top10_table))

write.csv(file="4_top10_marker_genes.csv",top10_table,row.names=F)

n=20

cutoff <-ifelse(min(table(sample.markers$cluster))>n,n,min(table(sample.markers$cluster)))

topN <- sample.markers %>% group_by(cluster) %>% top_n(n = cutoff, wt = avg_log2FC)

write.csv(file = paste0("4_top",cutoff,"_cell_markers.csv"),topN,row.names=F)

topN_table=unstack(topN, gene ~ cluster)

names(topN_table)=gsub("X","cluster",names(topN_table))

write.csv(file=paste0("4_top",cutoff,"_marker_genes.csv"),topN_table,row.names=F)

\###

pdf('4_classical_markers_featureplot-ACO2.pdf',width = 5,height = 3)

FeaturePlot(obj.combined, features = c("ACO2"),min.cutoff = "q9")

dev.off()

\###

new.cluster.ids <- c("HCC","HCC", "HCC","Myeloid Derived Cells","Endothelial Cells",

"HCC","Cancer Associated Fibroblasts","Other immune cells", "HCC")

names(new.cluster.ids) <- levels(obj.combined)

obj.combined <- RenameIdents(obj.combined, new.cluster.ids)

pdf('4_cluster_annotations.pdf',width = 10,height = 7)

DimPlot(obj.combined, reduction = "umap", label = TRUE, pt.size = 0.5) + NoLegend()

dev.off()

\###

#sample.markers <- FindAllMarkers(obj.combined, only.pos = TRUE, min.pct = 0.25, logfc.threshold = 0.25)

top3 <- sample.markers %>% group_by(cluster) %>% top_n(n = 3, wt = avg_log2FC)

pdf('4_Heatmap_cluster_name.pdf', width = 16, height = 11)

DoHeatmap(obj.combined, features = top3$gene, size = 3.5, angle = -75,hjust = 1)

dev.off()

\###

write.csv(file="HCC_genes.csv",allHCCcell@assays$RNA@data)

pdf('ACO2-HCC.pdf', width = 8, height = 5)

allHCCcell<-subset(obj.combined,ident=c("HCC"))

DimPlot(allHCCcell)

dev.off()

pdf('HCC2.pdf', width = 8, height = 5)

Idents(allHCCcell)<-'HCC'

DimPlot(allHCCcell)

dev.off()

pdf('ACO2 in HCC.pdf', width = 6, height = 5)

FeaturePlot(allHCCcell,features = "ACO2")

dev.off()

highCells=colnames(subset(x = allHCCcell, subset =rna_ACO2 > 0, slot = 'data'))

highORlow=ifelse(colnames(allHCCcell) %in% highCells,'high','low')

table(highORlow)

allHCCcell@meta.data$highORlow=highORlow

markers2 <- FindMarkers(allHCCcell, ident.1 = "high",

group.by = 'highORlow',

assay='RNA',slot='data')

head(x = markers2)

write.csv(markers2,file="HCC_ACO2_all.csv")
