# Supplementary material for: Pan-Cancer analysis shows that ACO2 is a potential prognostic and immunotherapeutic biomarker for multiple cancer types including hepatocellular carcinoma
Source: Front Oncol. 2022 Nov 30;12:1055376. doi: 10.3389/fonc.2022.1055376 (PMC9748622; doi:10.3389/fonc.2022.1055376)
Supplement: Supplementary file 1 [file DataSheet_1.zip › Original data/R package-pROC.doc]

library(tidyverse)

library(pROC)

library(ggplot2)

library(reshape2)

library(rstatix)

\# data <- dat

data <- read.table("~/file.txt", header = T)

data$outcome <- factor(data$outcome, levels = c("group1", "group2"))

head(data)

\#	outcome	a	b	c

\# 1 group1 1.585855 1.1742805 2.674788

\# 2 group1 2.205293 0.8619279 2.003079

\# 3 group1 2.199554 2.3158722 1.281605

\# 4 group1 1.241118 1.5746377 1.866428

\# 5 group1 2.016992 1.9533336 1.847221

\# 6 group1 2.391271 1.0891951 2.149648

data2 <- gather(data, key = "x", value = "value", -outcome)

data3 <- data2 %>%

group_by(outcome, x) %>%

get_summary_stats(value)

data3

\# outcome x	variable	n	min	max median	q1	q3	iqr	mad mean	sd	se	ci

\# 1 group1 a	value	40 0.586 2.44 1.45 1.12	1.94 0.822 0.631 1.51 0.555 0.088 0.177

\# 2 group1 b	value	40 0.556 2.50 1.8	1.14	2.21 1.07 0.777 1.65 0.619 0.098 0.198

\# 3 group1 c	value	40 1.05	2.95 2.07 1.77	2.44 0.665 0.495 2.04 0.527 0.083 0.169

\# 4 group2 a	value	32 0.129 1.99 0.996 0.623 1.50 0.878 0.633 0.998 0.554 0.098 0.2

\# 5 group2 b	value	32 0.022 1.96 0.779 0.325 1.18 0.86 0.624 0.843 0.572 0.101 0.206

\# 6 group2 c	value	32 0.03	1.89 0.923 0.421 1.39 0.969 0.706 0.932 0.546 0.097 0.197

roc1 <- roc(response = data$outcome, predictor = data$a)

\# Call:

\#	roc.default(response = data$outcome, predictor = data$a)

\#

\# Data: data$a in 40 controls (data$outcome group1) > 32 cases (data$outcome group2).

\# Area under the curve: 0.7328

roc2 <- roc(response = data$outcome, predictor = data$b)

\# Call:

\#	roc.default(response = data$outcome, predictor = data$b)

\#

\# Data: data$b in 40 controls (data$outcome group1) > 32 cases (data$outcome group2).

\# Area under the curve: 0.8234

roc3 <- roc(response = data$outcome, predictor = data$c)

\# Call:

\#	roc.default(response = data$outcome, predictor = data$c)

\#

\# Data: data$c in 40 controls (data$outcome group1) > 32 cases (data$outcome group2).

\# Area under the curve: 0.9242

ci.auc(roc1)

\# 95% CI: 0.6171-0.8485 (DeLong)

ci.auc(roc2)

\# 95% CI: 0.7303-0.9165 (DeLong)

ci.auc(roc3)

\# 95% CI: 0.8679-0.9805 (DeLong)

coords(roc1, x = "best", ret="all")

\#	threshold specificity sensitivity accuracy tn tp fn fp	npv	ppv	fdr fpr	tpr tnr	fnr

\# threshold 1.115538	0.75	0.625 0.6944444 30 20 12 10 0.7142857 0.6666667 0.3333333 0.25 0.625 0.75 0.375

\#	1-specificity 1-sensitivity 1-accuracy	1-npv	1-ppv precision recall youden closest.topleft

\# threshold	0.25	0.375 0.3055556 0.2857143 0.3333333 0.6666667 0.625 1.375	0.203125

roc.test(roc1, roc2, reuse.auc=FALSE, method = "delong")

\# DeLong's test for two correlated ROC curves

\#

\# data: roc1 and roc2

\# Z = -1.2406, p-value = 0.2148

\# alternative hypothesis: true difference in AUC is not equal to 0

\# sample estimates:

\# AUC of roc1 AUC of roc2

\#	0.7328125	0.8234375

plot.roc(roc1)

data1 <- data.frame(group = "a",

x = 1-roc1$specificities,

y = roc1$sensitivities)

data1 <- data1[order(data1$x, data1$y),]

tmp <- data.frame(group = "b",

x = 1-roc2$specificities,

y = roc2$sensitivities)

tmp <- tmp[order(tmp$x, tmp$y),]

data1 <- rbind(data1, tmp)

data1$group <- factor(data1$group, levels = c("a", "b"))

ggplot() +

geom_line(data = data1, aes(x = x, y = y, colour = group)) +

labs(x = "1-Specificity (FPR)", y = "Sensitivity (TPR)")
